# Potential Prophylactic Treatments for COVID-19

**DOI:** 10.3390/v13071292

**Published:** 2021-07-02

**Authors:** Noam Ben-Zuk, Ido-David Dechtman, Itai Henn, Libby Weiss, Amichay Afriat, Esther Krasner, Yoav Gal

**Affiliations:** 1 Chemical, Biological, Radiological and Nuclear Defense Division, Ministry of Defense, HaKirya, Tel-Aviv 61909, Israel; noambenzuk@yahoo.com (N.B.-Z.); itaihenn98@gmail.com (I.H.); nbc_pd@mod.gov.il (L.W.); 2 The Israel Defense Force Medical Corps, Tel Hashomer, Military Post 02149, Israel; ido.dechtman@gmail.com; 3 Pulmonology Department, Edith Wolfson Medical Center, 62 Halochamim Street, Holon 5822012, Israel; 4 Department of Molecular Cell Biology, Weizmann Institute of Science, Rehovot 7610001, Israel; afriat.a@gmail.com; 5 Israel Institute for Biological Research, Ness-Ziona 76100, Israel

**Keywords:** SARS-CoV-2, COVID-19, treatment, prophylaxis, pre-exposure, post-exposure, repurposed drugs

## Abstract

The World Health Organization declared the SARS-CoV-2 outbreak a Public Health Emergency of International Concern at the end of January 2020 and a pandemic two months later. The virus primarily spreads between humans via respiratory droplets, and is the causative agent of Coronavirus Disease 2019 (COVID-19), which can vary in severity, from asymptomatic or mild disease (the vast majority of the cases) to respiratory failure, multi-organ failure, and death. Recently, several vaccines were approved for emergency use against SARS-CoV-2. However, their worldwide availability is acutely limited, and therefore, SARS-CoV-2 is still expected to cause significant morbidity and mortality in the upcoming year. Hence, additional countermeasures are needed, particularly pharmaceutical drugs that are widely accessible, safe, scalable, and affordable. In this comprehensive review, we target the prophylactic arena, focusing on small-molecule candidates. In order to consolidate a potential list of such medications, which were categorized as either antivirals, repurposed drugs, or miscellaneous, a thorough screening for relevant clinical trials was conducted. A brief molecular and/or clinical background is provided for each potential drug, rationalizing its prophylactic use as an antiviral or inflammatory modulator. Drug safety profiles are discussed, and current medical indications and research status regarding their relevance to COVID-19 are shortly reviewed. In the near future, a significant body of information regarding the effectiveness of drugs being clinically studied for COVID-19 is expected to accumulate, in addition to information regarding the efficacy of prophylactic treatments.

## 1. Introduction

SARS-CoV-2 belongs to the *Coronaviridae* family of single-stranded RNA viruses, with a crown-like structure dictated by the spatial conformation of its spike proteins [1]. Coronavirus Disease 2019 (COVID-19), the disease resulting from exposure to this pathogen, is characterized by a pulmonary pathology which can progress to acute respiratory distress syndrome (ARDS), respiratory failure, and death [2]. The virus enters cells by binding to the angiotensin converting enzyme-2 (ACE-2) receptor expressed on subpopulations of pulmonary epithelial cells (type II epithelial cells), after which it is primed (cleavage of the spike protein) by the transmembrane serine 2 protease (TMPRSS2) as part of the fusion process with the cell membrane, and then internalized. ACE2 internalization and activation of the immune system [3] trigger pulmonary inflammation, characterized by increased levels of pro-inflammatory markers, such as interleukin-6 (IL-6) and C-reactive protein (CRP). This upregulation of inflammatory mediators progresses in some patients to a “cytokine storm” [4]. In severe cases, vascular damage, manifested by endothelial cell damage and hypercoagulation, is observed [5].

Severe morbidity and disease-related mortality are generally observed among patients aged 65 and above, as well as in individuals with comorbidities, e.g., obesity, hypertension, diabetes, heart disease, and others (herein, “at-risk population”) [6]. In addition, individuals who are in close contact with COVID-19 patients (i.e., healthcare professionals, social workers, and first-responders), as well as people living in densely populated areas or with a lifestyle that includes routine congregation (e.g., boarding schools and military units), are at higher risk of contracting the virus.

Currently, the only effective means to curb the COVID-19 pandemic is via widespread anti-SARS-CoV-2 vaccination. There are several vaccines available following emergency use authorization. However, it will take a long time until these vaccines are available for mass population vaccination on a global scale. Furthermore, vaccination is contraindicated in specific subpopulations (i.e., subjects suffering from severe allergic reaction to vaccine components) and little is known about the vaccines’ long-term efficacy. Logistic issues, such as storage, transportation, handling, and administration, also pose significant challenges. Therefore, other countermeasures are urgently required.

Apart from supportive treatment, which aims to ameliorate symptoms, provide invasive and non-invasive respiratory support, and prevent secondary complications, attempts are being made to establish a medical response via passive immunizations and pharmaceutical treatments. To date, few drugs have proven useful in treating COVID-19, most of which are administered in advanced COVID-19 disease states, i.e., steroids [7].

Prophylactic treatment for infectious diseases involves drug administration as early as possible and is generally given to healthy individuals [8]. Prophylaxis can be given before (pre-exposure) or after (post-exposure) encountering the pathogen, but prior to symptoms onset. The earlier infectious diseases are treated with antimicrobials or immunomodulatory drugs (anti-inflammatories), the better the prognosis. This is due to the lower inoculum and levels of inflammatory components (e.g., cytokines and neutrophils) at early stages of infection [9,10].

This work will review the current knowledge on COVID-19 prophylactics.

## 2. Methods

This review focuses on drugs reported as potentially effective for COVID-19 in the media, journals, as well as in pre-clinical or in vitro studies.

We examined pharmaceuticals with known anti-viral effects, including repurposed drugs and antibacterial drugs. A list of all relevant clinical studies, registered at the NIH website (clinicaltrails.gov) was constructed. Our search methodology in the above websites used the keywords combination of the “name of the drug” + “COVID-19”, filtering out irrelevant results. We compared all candidates’ performances based on their safety, known long-term efficacy, prophylactic usage, anti-viral coverage, and their registered clinical trials’ preliminary results. This methodology led to a list of 11 relevant drug candidates; 9 of whom have registered prophylactic studies (Table 1). After assembling the information, we summarized educated suggestions.

Inclusion criteria: from the initial search, only the studies that followed the below criteria were included in our article:

1. The drug is clinically evaluated for COVID-19.
2. The drug possesses regulatory approval from the US Food and Drug Administration (FDA), European Medicines Agency (EMA), The Medicines and Healthcare Products Regulatory Agency (MHRA), or the Pharmaceuticals and Medical Devices Agency (PMDA).
3. The drug is evaluated for prophylactic use for COVID-19/the drug is used prophylactically for other medical conditions/there’s data about previous long-term usage.

## 3. Current Prophylaxis Options for COVID-19

The drugs described below are currently being tested in clinical trials as COVID-19 prophylactics or as therapeutics with potential prophylactic applications (Table 2). Current status and information regarding the drugs is summarized in Table 3. The presented drugs are categorized as either antivirals, repurposed drugs, or miscellaneous.

### 3.1. Antivirals

#### 3.1.1. Favipiravir

Favipiravir (T-705) is a prodrug which, after being metabolized, inhibits various viral RNA-dependent RNA polymerases (RdRps) [12]. SARS-CoV-2 RdRp activity has been found to be ten-fold higher than that of other viral RdRps; therefore, favipiravir is a good candidate for COVID-19 treatment [13]. Viruses shown to have in vitro susceptibility to this drug include polio, zika, western equine encephalitis (WEE), seasonal/pandemic influenza, rabies, ebola, arenaviruses [14], and Rift Valley fever [15]. Favipiravir has also demonstrated efficacy against pandemic influenza in a range of animal and clinical studies [16,17] and has been approved for clinical use in Japan to treat recurrent or pandemic flu. Moreover, favipiravir exhibits a very favorable clinical safety profile. Its main side effects include hyperuricemia, which is reversed upon discontinuation of treatment, and elevated liver enzyme level [18]. However, the drug is teratogenic and is therefore prohibited for use during pregnancy [19]. Prophylactic clinical use of favipiravir has been reported post-exposure to ebola [20] and rabies [21]. Owing to its broad activity against RNA viruses, favipiravir has been frequently suggested as a potential treatment for COVID-19, and has demonstrated a significant antiviral effect when administered to hamsters prior to SARS-CoV-2 infection (prophylaxis animal model) [22].

When favipiravir was tested in COVID-19 patients, viral clearance was shortened from 11 to 4 days, and 91% of the treated patients had improved pulmonary inflammation markers, compared to 62% in the control group [23]. In a randomized, comparative, open-label, multicenter, phase 3 clinical trial, the drug led to a significant shortening of clinical cure time in COVID-19 patients [24].

In the context of prophylaxis, a clinical study currently being conducted in Canada (NCT04448119, Phase 2) is assessing the efficacy of favipiravir treatment over 25 days in preventing infection in nursing homes (among the elderly, assisted-living patients, and healthcare professionals). Results obtained in another clinical trial indicated that early intervention with favipiravir is superior to late intervention in terms of viral clearance and time to defervescence, further supporting the notion of a potential benefit of prophylactic administration [25].

#### 3.1.2. Combined Antiretroviral Medications

##### Lopinavir/Ritonavir

Lopinavir/ritonavir (LPV/r) has been FDA-approved since 2000 as a combination treatment for human immunodeficiency virus (HIV), and has demonstrated safety in patients without comorbidities [26]. The drug combination is safe for use in pregnant women, newborns [27,28], and infants [29].

Following demonstration of favorable clinical responses when administered to SARS-infected patients [30,31], LPV/r has been recommended for treatment of SARS-CoV-2 [32,33]. A study aiming to assess the efficacy of LPV/r alone in COVID-19 patients found no difference between the treatment and control cohorts [34].

These findings have led to treatment regimen changes and to the recommendation of the World Health Organization against LPV/r treatment in COVID-19 patients [35]. A recently published work presenting a comparative analysis of the literature related to the drug combination and coronaviruses proposed that blood concentrations of the drug suitable for HIV patients are insufficient for SARS-CoV-2 viral loads [36]. If proven correct, prophylactic LPV/r treatment at the regimens currently recommended for HIV may be effective when administered pre-exposure or at the early onset of exposure, when lower drug concentrations are required for a virucidal effect due to low inoculum. Recently published results summarizing a clinical trial in COVID-19 patients (NCT04379245) suggested that LPV/r would not be an effective preventive treatment. However, as it was a small-scale observational study [37], further assessments will be necessary before reaching final conclusions.

Two ongoing Phase 3 clinical studies are assessing the efficacy of prophylactic pre- and post (ring vaccination)—exposure treatments with LPV/r—NCT04321174 (recruiting) and NCT04364022. An additional Phase 3 study is evaluating the beneficial effects of prophylactic LPV/r treatment among healthcare workers (NCT04328285).

##### Emtricitabine/Tenofovir

Emtricitabine/tenofovir is a combination therapy used to treat and prevent HIV. The drug combination is composed of nucleoside analogues that impair viral genome replication. The combination is registered for use in adults and children under 12 years of age, and has been found effective in reducing risk of infection in virus-negative adults and as a pre-exposure prophylactic.

HIV exploits RNA-dependent DNA polymerase (RdDp), while coronaviruses use, as mentioned, RdRp. However, in vitro studies have unexpectedly demonstrated that the drug effectively inhibits the SARS-CoV-2 RdRp as well [38]. In particular, emtricitabine/tenofovir combination (the combination in DESCOVY and TRUVADA) was shown to be a terminator for the SARS-CoV-2 RdRp catalyzed reaction [39]. In light of the safety profile and potential anti-SARS-CoV-2 effects, the emtricitabine/tenofovir drug combination is currently being tested as a COVID-19 prophylactic. An ongoing Phase 3 clinical study is assessing the efficacy of a 12 week prophylaxis emtricitabine/tenofovir regimen (NCT04334928) in healthcare workers in contact with COVID-19-confirmed patients in private hospitals in Spain. Several additional studies aiming to use this drug combination in a preventive manner (NCT04519125, NCT04405271) are registered for Phase 2/3 and Phase 3, respectively, in the NIH website (clinicaltrials.gov), but are not yet recruiting participants.

### 3.2. Repurposed Drugs

#### 3.2.1. Ivermectin

Ivermectin is an antiparasitic, FDA-approved (for adolescents as well as adults), inexpensive drug used for the long-term treatment of scabies, lice, river blindness, and other parasitic diseases. The drug is derived from *Streptomyces avermitilis* and is administered mainly via the oral route. Its main side effects at human dosages are eye redness and dry/burning sensations of the skin. More serious side effects are rare and include central nervous system (CNS) suppression due to potentiation of the gamma aminobutyric acid (GABA) neurotransmitter synapses.

Regarding its antiparasitic effect, the drug binds glutamate-gated chloride channels on nerve and muscle cells of invertebrates, leading to increased permeability of parasite membranes to chloride ions, subsequently resulting in cell hyperpolarization, paralysis, and death. Its antiviral activity may be achieved by its inhibition of importin α/β, which is responsible for viral entry into the nucleus [40,41]. Studies have shown that the drug has antiviral potential against chikungunya, yellow fever, West Nile, Venezuelan equine encephalitis virus (VEEV), dengue, and influenza. The drug is also being tested as a prophylactic treatment for malaria [42].

The drug potentially possesses a dual anti-SARS-CoV-2 effect, namely antiviral and anti-inflammatory. Regarding its antiviral effect, the drug has shown a significant anti-SARS-CoV-2 effect in vitro with a 5000-fold reduction in viral RNA in cells within 48 h of exposure. However, the dosage required to inhibit SARS-CoV-2 replication in cells is equivalent to 10,000-fold the dosage currently approved for human use [40]. Furthermore, plasma concentrations of the drug after oral administration are estimated to be too low to be effective in inhibiting viral replication [43,44]. Regarding its anti-inflammatory properties, ivermectin was shown to inhibit lipopolysaccharide (LPS)-induced prostaglandin E2 and nitric oxide production in cell culture [45]. In vivo, ivermectin inhibits the production of inflammatory cytokines (IL-6, IL-1β, TNFα) and improved survival in mice exposed to LPS [46]; it was also found to be an allosteric modulator of the α7 nicotinic acetylcholine receptor (an essential regulator of inflammation [47]) in hamsters [48]. There are currently many clinical studies testing ivermectin alone or in combination with other anti-COVID-19 treatments. A retrospective review of the medical files of COVID-19 patients in four Florida hospitals found a significant reduction in mortality rates among patients who received the drug [49]. A randomized, controlled, and double-blinded trial demonstrated a significantly shorter period of time to SARS-CoV-2 negativity in parallel to increased oxygen saturation, following intravenous administration of ivermectin. The efficacy of ivermectin was dose-dependent and no serious adverse events were reported [50].

Furthermore, several studies evaluating the potential of its prophylactic use demonstrated highly encouraging results. Prophylactic ivermectin treatment of healthcare workers in India was associated with a 73% reduction in COVID-19 infection rates [51].

The drug has also been tested for its post-exposure prophylactic effects and was found to prevent symptoms among COVID-19 household contacts (NCT04422561), where 7.4% of the subjects in the treated group developed symptoms, compared to 58.4% of those treated with placebo.

Nine additional clinical trials are registered in the NIH website, three of which were completed and one large-scale study (4257 subjects) is in course of its third phase (Table 3). In addition to the clinical evidence, it was suggested that countries with routine mass administration of prophylactic chemotherapy, including ivermectin, have a significantly lower incidence of COVID-19 [52].

#### 3.2.2. Interferons

Interferons (IFNs) are endogenous proteins secreted following the penetration of pathogens into the body, which activate the immune system [53] and serve as a standard treatment for a range of diseases, including viral diseases, i.e., hepatitis C virus (HCV) and hepatitis B virus (HBV) [54].

IFN-α is a key IFN used in antiviral treatment [55]. The drug affects cellular regulation and prevents viral replication, while activating the adenylate cyclase enzyme on membrane receptors, which activates intracellular antiviral processes in DNA and RNA virus-mediated infections. IFN-α treatment of mice exposed to bacterial lipopolysaccharide (LPS) was associated with increased survival of mice suffering from pulmonary damage (ARDS). The increased survival was associated with the delayed onset of damage caused by neutrophil infiltration into the lungs [56]. Various IFNs have demonstrated long-term prevention of various virus-related infections, such as rhinovirus, influenza, Middle East respiratory syndrome (MERS), and SARS [57,58]. An in vitro study demonstrated their efficacy against SARS at interferon concentrations similar to those found in human serum [59]. A non-human primate study showed that treatment with a IFN-α nasal spray might prevent or reduce the pathology caused by SARS [60]. Furthermore, a clinical trial evaluating the efficacy of a recombinant human IFN-α (rhIFN-α 2b)-containing spray given twice daily to over 14,000 participants found the drug effective in preventing respiratory diseases induced by influenza, RSV, and adenoviruses, with a highly favorable safety profile [61].

Adverse effects due to IFN- α have been described in many organ systems [62]. Many side-effects are dose-dependent. The most common are flu-like symptoms, hematological toxicity, elevated hepatic transaminases, nausea, fatigue, and psychiatric sequelae. A special consideration regarding IFN treatment for SARS-CoV-2 are the IFN autoantibodies formation, which were found in severe COVID-19 patients [63,64]. It should be mentioned, though, that, although this could potentially hamper IFN bioactivity and treatment outcome, as in the case of severe COVID-19 patients, the relevance of autoantibody formation during prophylactic treatment, namely in healthy or in pre-symptomatic subjects, is yet to be determined.

A significant volume of evidence has recently accumulated regarding the potential of IFN treatment of COVID-19. SARS-CoV-2 was shown to inhibit IFN production both in vitro (membrane protein (M)-dependent inhibition) and in vivo [65]. Thus, exogenous administration of IFNs may compensate for this insufficiency [66]. In addition, PEGylated human interferon lambda-1 (PEG-IFN-λ-1a) potently delayed SARS-CoV-2 replication in epithelial cells, and prophylactic (pre-exposure) or therapeutic administration significantly lowered pulmonary viral load in a mouse model [67]. Furthermore, it was suggested that administration of recombinant or PEGylated forms of IFN-λ suppresses viral replication while preventing the onset of a “cytokine storm” [68].

There are many clinical trials currently assessing the efficacy of multiple interferons (IFN-λ-1a, IFN-β-1b, IFN-β-1a, novaferon, IFN-α, and PEG-IFN-λ-1a) in COVID-19 patients. A non-controlled study conducted in Wuhan, China weighing the efficacy of IFN-α-2b in 77 patients found that the treatment led to a significant decline in viral load in the upper respiratory tract and in IL-6 and CRP levels [69], emphasizing the potential dual effect (anti-viral and anti-inflammatory) of this drug in the course of COVID-19 treatment.

Prophylactic IFN treatments are being assessed in ongoing clinical trials (all of which are recruiting subjects to the studies, see Table 3). A Phase 3 study (NCT04320238) is evaluating the prophylactic efficacy and safety of rhIFN-α-1b nasal drops in healthy medical workers [70]. Another Phase 3 clinical trial in Chile is evaluating the post-exposure prophylactic use of three subcutaneous injections of PEG-IFNβ-1a in household contacts (NCT04552379), and a Phase 2b study conducted in the USA is evaluating the efficacy of PEG-IFN-λ-1a as a post-exposure prophylactic in high-risk, non-hospitalized individuals following household exposure (NCT04344600). A Phase 3 clinical trial is evaluating pre- and post-exposure prophylactic uses of IFNα nasal drops in oncology patients in Australia (NCT04534725).

#### 3.2.3. Nitazoxanide

Nitazoxanide is an anti-protozoal drug belonging to the thiazolides family, which disrupts critical energy-generating pathways required for pathogen survival and replication. Nitazoxanide is a prodrug, which, after hydrolysis and conjugation, becomes an active metabolite [71].

The drug is orally administered, and has been cumulatively used by over 75 million patients across the globe [72]. Accumulated data has shown the drug to be very safe for use in humans. Furthermore, due to its low price, the drug can be affordable worldwide [73].

Nitazoxanide has demonstrated antimicrobial activity and is currently used as a broad-spectrum antiviral medication. It has been proven to be effective in chronic hepatitis patients and is also indicated for the treatment of influenza. The drug leads to reduced HIV replication and, in parallel, stimulates immune memory responses [74].

The antiviral mechanism of nitazoxanide is likely to work via activation of eukaryotic translation initiation factor 2α, which serves as an intracellular antiviral factor [75]. In coronaviruses, the drug also inhibits nucleocapsid (N) protein expression and viral replication. Moreover, studies performed in patients infected with MERS and other coronaviruses showed a reduction in proinflammatory cytokine expression [72]. Nitazoxanide inhibited IL-6 production in a mouse inflammation model [76]. Therefore, in addition to its antiviral effects, nitazoxanide may serve as an immunomodulatory drug and suppress the intensity of the “cytokine storm” in COVID-19 patients.

Preclinical and clinical trials are being performed to evaluate the efficacy of nitazoxanide against SARS-CoV-2 [72,77]. Encouraging results have been obtained in in vitro models, which showed a high correlation between the attainable maximum plasma concentrations (C_max_) and the dosage required to inhibit SARS-CoV-2 replication [78]. To date, several studies are being conducted in COVID-19 patients. In most of the studies, the drug is being combined with other drugs, but some are evaluating the efficacy of nitazoxanide as monotherapy in patients with mild-to-moderate diseases. Early administration of nitazoxanide to mild COVID-19 patients has recently been reported to reduce viral loads; no serious adverse events were reported [79](NCT04552483). Two Phase 3 studies are currently being conducted; one is aiming to assess prevention of outbreaks among healthcare workers in frequent contact with confirmed COVID-19 patients at early stages of the disease with mild symptoms (NCT04359680), and the other is evaluating post-exposure prophylaxis in nursing home residents (NCT04343248). A Phase 2 study (NCT04561063) is exploring the efficacy of nitazoxanide in preventing COVID-19 in healthcare workers at high risk of exposure. Another Phase 2 study (NCT04435314, not yet recruiting) plans to determine the effect of post-exposure nitazoxanide administration to volunteers who are at high-risk of infection. A Phase 4 study recruits volunteers in Argentina (NCT04788407).

#### 3.2.4. Bromhexine Hydrochloride

The mechanism of SARS-CoV-2 entry into lung epithelial cells involves binding the viral spike protein to the human ACE2 receptor, after which it is cleaved at two sites (which enables a conformational change and fusion) by the TMPRSS2 enzyme, as detailed above. Therefore, inhibition of this enzyme is an attractive target for COVID-19 treatment [3].

The role of TMPRSS2 in viral fusion with lung cells was previously demonstrated for SARS; it has been established that, in addition to its role in disease onset, it is involved in viral antibody evasion [80,81]. Furthermore, it has been proposed that TMPRSS2 inhibition by nafamostat is effective against MERS [82].

The expectorant drug bromhexine hydrochloride (HCl) has shown to inhibit TMPRSS2 (repurposed effect) [83] and has a favorable safety profile [83]. Additionally, it is low-priced and requires no prescription in many countries. Although preliminary data suggest that bromhexine HCl mucolytic doses are sub-optimal for the treatment of SARS-CoV-2 [84], there is some encouraging, though inconclusive, evidence for its repurposing potential for COVID-19 treatment [85,86].

Two clinical trials are evaluating the drug’s efficacy as COVID-19 prophylaxis for healthcare workers; one is an ongoing Phase 1 clinical trial assessing oral administration (8 mg three times daily for two months, NCT04340349), and another is a small-scale trial in 50 subjects, which was completed (NCT04405999). Results are anticipated.

### 3.3. Miscellaneous

#### 3.3.1. Doxycycline

Doxycycline is a tetracycline antibacterial isolated from *Streptomyces aureofaciens* used to treat a broad range of infections. The drug is chiefly bacteriostatic and employs its antimicrobial effect via protein synthesis inhibition [87]. Its side effects include developing a rash, fever, lymph node swelling, flu-like symptoms, and yellow shading of the skin and eyes. In addition, doxycycline intake is associated with photosensitivity and may cause diarrhea. More severe side effects are rare. The drug is contraindicated during pregnancy (after the 18th week) and in children under eight years of age (except for emergencies) [88].

Doxycycline has been found effective against viral infections such as HIV and West Nile [89]. In a study that tested the effect of antibiotics on dengue-virus progression, doxycycline significantly inhibited the viral serine protease enzyme and significantly reduced viral replication and invasion into cells [90]. Doxycycline was also proven to reduce neurological deficits in a Zika virus mouse model [91]. Similarly, studies have suggested that tetracyclines may inhibit replication of single-stranded RNA viruses [92], including SARS-CoV-2, both in terms of cell entry and viral replication [93].

Doxycycline inhibits matrix metalloproteinases (MMPs), and, as coronaviruses exploit MMPs for a range of their essential activities (replication, cell infection, and survival), the drug may have high efficacy against SARS-CoV-2 [94]. According to other studies, doxycycline may delay COVID-19 progression via anti-inflammatory activities, including regulating the NFκB pathway and inhibition of proinflammatory cytokine levels (IL-6, IL-1β, TNFα), measured during ARDS in severely ill COVID-19 patients [95]. Earlier studies demonstrated the effectiveness of chemically modified tetracyclines against SARS, where septic shock and ARDS development were prevented [96].

Therefore, doxycycline treatment of COVID-19 patients may be effective. To date, there are several clinical trials registered on the NIH website testing its effectiveness against COVID-19. These studies are in Phases 2, 3, and 4. Although only one study evaluates doxycycline as a prophylactic agent for COVID-19 (NCT04584567, recruiting for phase 3), the drug has a prophylaxis indication, for example, in malaria. Moreover, doxycycline is administered for prolonged durationa as a post-exposure therapeutic against Q fever caused by *Coxiella burnetti* [97], indicating broad safety margins. Therefore, doxycycline may be a suitable prophylactic (primarily post-exposure) agent against COVID-19. Supporting the potential benefit of doxycycline prophylaxis for COVID-19, early treatment in high-risk patients with moderate-to-severe COVID-19 infections in non-hospital settings was associated with early clinical recovery, decreased hospitalization, and decreased mortality [98]. Several case reports may also support the notion of a beneficial effect of doxycycline preventive treatment. In a series of four patients with COVID-19 infection and known high-risk pulmonary disease who were placed on standard doses of doxycycline as monotherapy for a course between 5 and 14 days, a rapid clinical improvement was recorded with no safety issues noted [99]. Nevertheless, after reviewing the interim analysis of the doxycycline arm of the PRINCIPLE trial held in the UK, it was concluded that there is no beneficial effect in patients aged over 50 who are treated at home with doxycycline during the early stage of COVID-19 [100].

#### 3.3.2. Famotidine

Famotidine is a histamine-2 receptor antagonist used to treat heartburn, gastroesophageal reflux, and peptic (stomach and duodenum) ulcers. Side effects generally include fatigue, headaches, abdominal pain, and diarrhea. Severe side effects are relatively rare [101]. Laboratory studies have found the drug to inhibit HIV replication [102]. A bioinformatic study identified the drug as a candidate inhibitor of 3-chymotrypsin-like protease (3CLpro) involved in SARS-CoV-2 replication [103]. Apart from 3CLpro inhibition, famotidine has been suggested to impart an immunomodulatory effect, since lower ferritin levels were measured in patients receiving the drug [104].

A retrospective cohort study found that COVID-19 subjects who had received famotidine (at any dosage or administration route) within 24 h of hospitalization had approximately 2-fold lower mortality and intubation rates. The treatment was seemingly given before the onset of symptoms as a treatment for heartburn, and, in most cases, it was given prior to SARS-CoV-2 infection. Famotidine self-administered by 10 non-hospitalized COVID-19 patients was found safe at high dosages, and was associated with significant self-reported symptomatic improvement [105]. To date, several clinical trials registered on the NIH website are enrolling participants to evaluate the efficacy of famotidine in COVID-19 patients, including outpatients.

In light of the high safety profile, potential antiviral effects, clinical findings mentioned above, and the possibility of long-term treatment, the drug may be suitable as a COVID-19 prophylactic. It should be mentioned, though, that several studies demonstrated conflicting data regarding famotidine efficacy in COVID-19 patients [105,106]; therefore, its clinical impact should be further validated in randomized controlled trials, particularly for prophylactic use.

#### 3.3.3. Nitric Oxide

Nitric oxide (NO) is a neurotransmitter that interacts with many molecules, including DNA, proteins, and thiol-containing molecules. NO plays a critical role in immune system activation [107], where its derivatives (reactive nitrogen species) are generated by macrophages and other cells in response to cytokines and microbial substances. Moreover, all isoforms of the NO synthase (NOS) enzyme play a central role in the innate and adaptive immune responses.

As a smooth muscle relaxant, leading to pulmonary vasodilation, inhaled NO (iNO) is used for long-term treatment of pulmonary hypertension-associated hypoxia in infants. In a recent clinical trial conducted in several hospitals in Israel which assessed the efficacy of NO treatment in infants suffering from acute viral bronchitis [108], there were no reports of severe side effects, even after high dosage treatment (160 ppm) as an adjunct to oxygen (O_2_). O_2_ saturation in infants treated with iNO was higher and their recovery time was shorter. Safety was also demonstrated following long-term treatment (7–12 months) of adults with pulmonary hypertension [109].

Studies have shown that iNO may be an effective antiviral treatment. The literature shows that NO and its derivatives inhibit inflammatory processes via IFNγ-mediated mechanisms [110]. Furthermore, inverse correlations between NO levels and activity of viral enzymes (reductase and protease) that play essential roles in viral replication and activity have been reported [111,112]. Its antiviral effects against influenza viruses (influenza A & B), coronaviruses, vaccinia virus, herpes simplex type 1, and ectromelia have been shown in laboratory studies [113,114,115]. A small-scale study assessing the efficacy of NO treatment in patients with SARS found it to reduce viral spread in the lungs and to increase O_2_ saturation [116]. The recently developed nitric oxide release solution (NORS, developed by SaNOtize), which enables a delayed release of NO through a nasal spray/wash/gargling solution, was tested in the University of Utah and was found to inhibit influenza A and SARS-CoV-2 in vitro.

To date, several clinical trials are assessing the efficacy of NO treatment in COVID-19 patients. A Phase 2/3 study (NCT04842331) is recruiting 600 subjects to evaluate the efficacy of RESP301 (NO generating solution) as a post-exposure prophylaxis in household residents in the UK. Another study (NCT04312243, Phase 2) is assessing the prophylactic efficacy of NO among medical workers in close contact with confirmed SARS-CoV-2-positive patients. Additional study is assessing the efficacy of NO nasal spray (GLS-1200) given 3 times daily for 4 weeks to prevent SARS-CoV-2 infection among healthcare workers in close contact with confirmed COVID-19 patients (NCT04408183, Phase 2). An ongoing Phase 2 study (NCT04337918) is assessing NORS use as a prophylactic for individuals who came into close contact with confirmed SARS-CoV-2-positive patients. A small Phase 2 study (NCT04858451, not yet recruiting) will be conducted in order to evaluate the efficacy of RESP301 in patients at risk of viral infection (COPD and bronchiectasis patients).

#### 3.3.4. Colchicine

Colchicine is a drug orally or intravenously administered as a long-term prophylaxis against gout, familial Mediterranean fever (FMF), and recurrent pericarditis. The drug inhibits microtubule polymerization, disrupting cell division [117]. Moreover, the drug inhibits neutrophil migration to sites of inflammation, thereby serving as an anti-inflammatory [118]. Colchicine use in pregnancy and breastfeeding is controversial with an FDA class C recommendation, although recent data suggests a more lenient approach regarding its usage during pregnancy and breastfeeding [119,120,121]. Side effects include a variety of symptoms ranging from mild and frequent (e.g., gastrointestinal symptoms such as vomiting, nausea, and abdominal pain) to rare and serious (e.g., hematological dyscrasia, myalgia, neuropathy, confusion, and convulsions) [117,122,123,124,125].

The drug is absorbed by immune cells, granulocytes, and monocytes within 24–72 h of oral administration, inducing an anti-inflammatory effect. It has been recently shown that the drug also inhibits NLRP3 inflammasome [126]. This finding is of importance, since SARS-CoV-2 has been demonstrated to activate inflammatory processes by activation of NLRP3. Specifically, NLRP3 activation takes place early in the SARS-CoV-2 infection, initiating the “cytokine storm” [127].

Colchicine is currently being evaluated in clinical trials among COVID-19 patients. In particular, recent results obtained in 4159 non-hospitalized patients have associated colchicine use with reduced risk of death and hospitalization compared to placebo. Hospitalization rates were reduced by 25%, the need for mechanical ventilation by 50%, and deaths by 44%. Treatment was initiated within 24 h of symptom onset, and inclusion criteria included at least one significant risk factor [128]. Another Phase 3 study is assessing colchicine treatment of 954 confirmed SARS-CoV-2-positive non-hospitalized patients aged 60 years and above who were at risk of developing respiratory complications (NCT04416334). An additional Phase 2/3 study (NCT04492358) is underway in Spain to evaluate the efficacy of combining colchicine and prednisone for moderate/severe COVID-19 in a vulnerable population (geriatric hospital unit/transitional care center residents).

If those studies yield positive results, and the drug proves effective in adult patients, it will lay the foundation for more extensive studies to assess its prophylactic efficacy in COVID-19 patients, particularly in elderly populations at risk of complications and mortality, with emphasis on post-exposure prophylactic administration.

## 4. Discussion

To date, nearly 180 million people worldwide have been infected with SARS-CoV-2, with approximately four million deaths suffered. While several vaccines have been approved for emergency use, there is currently a severe shortage in their global supply. Additionally, vaccine efficacy is yet to be determined, in terms of longevity and coverage of emerging SARS-CoV-2 variants, current and future [129]. Furthermore, clinical vaccine studies in children and pregnant women are only just being conducted, currently leaving these populations unvaccinated. Likewise, severely immunocompromised patients cannot be effectively vaccinated, nor can the severely allergic. Hence, there is an urgent need for applicable treatment modalities, particularly pharmaceutical drugs.

The goal of this review is to highlight the potential for prophylactic efficacy of several approved and safe drugs. Favipiravir, IFNs, ivermectin, nitazoxanide, bromhexine HCl, and NO seem to be promising candidates. In particular, Ivermectin, which dramatically reduced COVID-19 infection rates or symptoms when administered as pre-exposure or post-exposure prophylaxis, respectively. In addition to ivermectin, early treatment of at-risk hospitalized COVID-19 patients with colchicine was associated with a significant reduction in the risk of both death and the need for mechanical ventilation, and its efficacy is currently being evaluated in non-hospitalized patients aged 60 years and above. Thus, colchicine also shows high potential for prophylactic treatment (post-exposure). Lastly, early intervention with doxycycline was shown to be beneficial in a clinical trial conducted in the USA. Nevertheless, as mentioned, current reports from the PRINCIPLE trial in the UK have shown conflicting outcomes. A possible explanation for this discrepancy is the earlier drug administration in the USA trial (up to 12 h of symptom onset, in comparison to 14 days within symptoms onset in the UK trial), further supporting its prophylactic rather than therapeutic potential. Additionally, the majority of the patients in the USA trial received zinc and calcium supplements in addition to doxycycline [98]. In this regard, it would be interesting to follow the consequences of the trial (NCT04584567) currently evaluating the prophylactic potential of doxycycline co-administered with zinc. Regarding IFNs, although seemingly efficacious and safe, their high cost (Table 2) limits their potential usage for widespread prophylaxis.

It should be mentioned that combination treatments, especially with safe drugs possessing different mechanisms of action, could be of great advantage. For example, early triple antiviral therapy (LPV/r, IFN-β-1b and ribavirin) was safe and superior to LPV/r alone in alleviating symptoms and shortening viral shedding duration and hospitalization in patients with mild-to-moderate COVID-19 symptoms [130]. Furthermore, co-incubation of remdesivir and ivermectin in cell culture conferred synergistic anti-SARS-CoV-2 effects [131]. Additionally, several clinical trials are currently evaluating concomitant administration of two drugs from the list depicted in this manuscript, i.e., nitazoxanide and ivermectin (NCT04360356), nitazoxanide, and favipiravir (NCT04918927, early intervention), as well as ivermectin and doxycycline (NCT04523831 and NCT04729140—completed and phase 4, respectively). If found beneficial, it is worth evaluating these combinations as prophylactic treatments, depending on the combinations’ safety profiles.

Major drawbacks of some drugs tested for COVID-19 are their extensive side effects, e.g., chloroquine/hydroxychloroquine [132], and absorption issues which require intravenous administration, e.g., remdesivir [133,134]. Inhalation or intranasal administration routes of these drugs may overcome, at least partially, these limitations, improving their suitability for prophylactic usage. Indeed, some clinical trials are evaluating the therapeutic impact of inhaled hydroxychloroquine (NCT04477083, NCT04461353) and remdesivir (NCT04480333). Further developments regarding administration routes should be monitored.

Many of the drugs presented in this review can be characterized as broad-spectrum antivirals/antimicrobials (favipiravir, interferons, ivermectin, nitazoxanide, bromexine HCl, and NO). Extensive antiviral and antimicrobial coverage, including against influenza, could be a dual advantage, particularly during the winter season when healthcare system burdens are particularly high.

In addition to small molecule drugs for COVID-19 prophylaxis, several additional approaches not reviewed here may be relevant for prophylaxis, as detailed below.

**Vitamins, dietary supplements, and antioxidants** are being assessed as both preventive and adjunct-therapeutic agents. Due to their high safety profiles and indirect beneficial effects (primarily in the elderly population, who often suffer from dietary deficits or vitamin deficiencies), recommendation of usage of these products, which are being tested in clinical trials, should be considered. Moreover, due to frequent lockdowns, rates of malnutrition and vitamin D insufficiency may have increased. These supplements were extensively reviewed elsewhere [133,134] and therefore were not included in this review.

**Antibody-based treatment** may be a promising strategy for both pre- and post-exposure prophylaxis [132,135]. Yet, this treatment is costly compared to small molecule-based treatment, rendering it unsuitable for mass administration. Furthermore, the approach is time-consuming, requiring a parenteral route of administration and medical surveillance by highly trained medical personnel in dedicated medical facilities. This approach may also be ineffective against highly mutated variants, particularly when monoclonal antibodies are used [136].

**Long-term boosting of innate immune response** using vaccines which are not directed against SARS-CoV-2 (immunostimulants) are under extensive clinical evaluation for COVID-19 prevention. Decreased immune functioning may lead to a loss of control of viral replication and, as a result, increased disease severity. Long-term boosting of innate immunity by various types of immunization strategies may cause a nonspecific stimulation of the immune system. In many cases, the immediate and effective activation of the innate immune system is sufficient to cope with the invading pathogen, even without activating adaptive immunity. Long-term changes manifested by enhanced innate immune activity have been demonstrated in individuals who received live vaccines [137]. In light of these findings, various live attenuated vaccines designed originally to prevent infection with a pathogen of interest (other than SARS-CoV-2) are being clinically tested for the treatment of COVID-19. This includes the Bacillus Clamette-Guérin (BCG) vaccine which effectively protects against various infectious diseases, as well as the VPM1002 (a genetically engineered BCG strain with higher immunogenicity) [138], the oral polio vaccine, the tuberculosis vaccine [139], and the varicella-zoster vaccine (NCT04523246), which are all being clinically tested for the prevention or alleviation of COVID-19.

In summary, clinically approved drugs with well-established and favorable safety profiles repurposed as a COVID-19 prophylaxis should be considered for at-risk individuals, as well as first responders and medical teams. It is imperative to keep abreast on this topic with clinical research developments across the globe.

## Figures and Tables

**Table 1:** Search outcomes summary.

Drug	All Clinical Trials	Prophylactic Clinical Trials
Favipiravir	38	1
LPV/r	38	4
Emtricitabine/Tenofovir	7	3
Ivermectin	68	10
Interferons	40	5
Nitazoxanide	28	5
Bromhexine hydrochloride (HCl)	6	2
Doxycycline	14	1
Famotidine	8	0
Nitric Oxide (NO)	22	5
Colchicine	26	0

**Table 2:** Candidate SARS-CoV-2 prophylactic drugs currently in clinical trials.

Drug		Safety	Cost ^2^	Long-Term Treatment	COVID-19 Clinical Trial		Administration ^3^
			(Price per Dose in USD, Single Dose)		Age +65	Prophylaxis	
Antiviral	Favipiravir	++++	N.A. ^4^	25 days (COVID-19)	+	+	per os (P.O.)
	LPV/r	+++	3.9–4.6 (400–100 mg/5 mL)	Unlimited		+	P.O.
	Emtricitabine/ tenofovir	+++	36.02–49.32 (300 mg)	Unlimited (HIV) 12 weeks (COVID-19)		+	P.O.
Repurposed Drugs	Ivermectin	+++	3.95 (3 mg)	15–17 year (up to ×2 a year)		+	P.O./intravenous (I.V.)
	Interferons	++++	9274 (4 mL) ^5^ 6985 (0.3 mg) ^6^	28 days (COVID-19)		+	Mucosal, Parenteral
	Nitazoxanide	++++	140.8 (500 mg) ^7^	3–24 months	+	+	P.O.
	Bromhexine HCl	++++	0.1–0.6 (8 mg)	2 months (COVID-19)		+	P.O.
Miscellaneous	Doxycycline	+++	0.6–2 (50/100 mg)	Months			P.O./I.V.
	Famotidine	++++	0.08–2.04 (20 mg)	Unlimited		+/−	P.O./I.V.
	Nitric Oxide (NO)	++++ ^1^	N.A.	5–17 months 4 weeks (COVID-19)		+	Inhalation, Topical ^3^
	Colchicine	+++	2.24–2.55 (0.6 mg)	Unlimited 21 days (COVID-19)	+		P.O.

^1^ NO release solutions and nasal drops are associated with high safety margins; ^2^ According to online price evaluation at drugs.com [11]; ^3^ Nasal spray/wash/gargling; ^4^ Not available; ^5^ IFN-β-1a; ^6^ IFN-β-1b; ^7^ Cheaper generic forms are available. “++++” is considered to have a highly favorable safety profile; “+++” is considered to have a favorable safety profile.

**Table 3:** Clinical trials status of candidate for SARS-CoV-2 prophylactic drugs *.

Drug	NCT	Phase	Participants	Country	Remarks
Ivermectin	NCT04832945	Completed	713	Dominican Republic	
	NCT04668469	Completed	600	Egypt	
	NCT04425850	Completed	229	Argentina	Positive results
	NCT04446104	3	4257	Singapore	
	NCT04891250	4	800	Zambia	Not yet recruiting
	NCT04527211	3	550	Colombia	Not yet recruiting
	NCT04894721	2/3	750	Argentina	Recruiting
	NCT04422561	2/3	340	Egypt	
	NCT04701710	1/2	300	Argentina	+Iota-carrageenan
	NCT04384458	N.A.	400	Brazil	Recruiting
Nitazoxanide	NCT04788407	4	456	Argentina	Recruiting
	NCT04359680	3	1407	USA	
	NCT04343248 ^1^	3	800	USA	
	NCT04561063	2	1950	South Africa	Recruiting
	NCT04435314	2	200	Brazil	Not yet recruiting
Emtricitabine/Tenofovir	NCT04334928	3	4000	Spain	Recruiting
	NCT04405271	3	1378	Argentina	Not yet recruiting
	NCT04519125	2/3	950	Colombia	Not yet recruiting
LPV/r	NCT04328285	3	1200	France	
	NCT04364022	3	326	Switzerland	
	NCT04321174	3	1220	Canada	Recruiting
	NCT04251871	N.A.	150	China	Recruiting ^6^
Interferons	NCT04534725	3	2282	Australia ^5^	Recruiting
	NCT04320238	3	2944	China	Recruiting
	NCT04552379	3	1240	Chile	Recruiting
	NCT04344600	2	164	USA	Recruiting
	NCT04251871	N.A.	150	China	Recruiting ^6^
Doxycycline	NCT04584567 ^3^	3	1100	Tunisia	Recruiting
Nitric Oxide (NO)	NCT04842331	2/3	600	UK	Recruiting
	NCT04408183	2	225	USA	Recruiting
	NCT04337918	2	143	Canada	
	NCT04858451 ^2^	2	150	UK	Not yet recruiting
	NCT04312243	2	24	USA	
Favipiravir	NCT04448119	2	760	Canada	
Bromhexine HCl	NCT04405999	Completed	50	Russia	
	NCT04340349 ^4^	1	214	Mexico	

* Drugs were ordered according to available data volume and clinical trial status; ^1^ Post-exposure prophylaxis of COVID-19 and other viral respiratory illnesses in elderly residents of Long-Term Care Facilities (LTCF); ^2^ In COPD and bronchiectasis patients; ^3^ Combined with zinc; ^4^ Combined with hydroxychloroquine; ^5^ In cancer patients; ^6^ Not the main subject of the study. It is a part of the standard of care treatment.
